# 

**Published:** 1900-08-18

**Authors:** 


					0ARE?RY'S ?f H I* CADBURY'S
COCOA _ _ ^ __
ABSOLUTELY PUU. COCOA
NO DRUGS OR ALKALIES USED.
No. 72p. Vol. XXYIII. Saturday,
August 18, 1900.
[SubsoriptwnTerms.'J pRICE TWOPENCE.

				

